# Atrioventricular Septal Defect: What Is in a Name?

**DOI:** 10.3390/jcdd8020019

**Published:** 2021-02-15

**Authors:** Michael Rigby

**Affiliations:** MD FRCP FRCPCH, Royal Brompton Hospital, London SW3 6NP, UK; M.Rigby@rbht.nhs.uk

**Keywords:** atrioventricular septal defect, AVSD, atrioventricular junction, bridging leaflets, nomenclature, classification

## Abstract

Robert Anderson has made a huge contribution to almost all aspects of morphology and understanding of congenital cardiac malformations, none more so than the group of anomalies that many of those in the practice of paediatric cardiology and adult congenital heart disease now call ‘Atrioventricular Septal Defect’ (AVSD). In 1982, with Anton Becker working in Amsterdam, their hallmark ‘What’s in a name?’ editorial was published in the *Journal of Thoracic and Cardiovascular Surgery*. At that time most described the group of lesions as ‘atrioventricular canal malformation’ or ‘endocardial cushion defect’. Perhaps more significantly, the so-called ostium primum defect was thought to represent a partial variant. It was also universally thought, at that time, that the left atrioventricular valve was no more than a mitral valve with a cleft in the aortic leaflet. In addition to this, lesions such as isolated cleft of the mitral valve, large ventricular septal defects opening to the inlet of the right and hearts with straddling or overriding tricuspid valve were variations of the atrioventricular canal malformation. Anderson and Becker emphasised the differences between the atrioventricular junction in the normal heart and those with a common junction for which they recommended the generic name, ‘atrioventricular septal defect’. As I will discuss, over many years, they continued to work with clinical cardiologists and cardiac surgeons to refine diagnostic criteria and transform the classification and understanding of this complex group of anomalies. Their emphasis was always on accurate diagnosis and communication, which is conveyed in this review.

## 1. Introduction

Robert Anderson has made a huge contribution to almost all aspects of morphology and understanding of congenital cardiac malformations, none more so than the group of anomalies that many of those in the practice of paediatric cardiology and adult congenital heart disease now call ‘Atrioventricular Septal Defect’ (AVSD). In 1982, with Anton Becker working in Amsterdam, their hallmark ‘What’s in a name?’ editorial was published in the Journal of Thoracic and Cardiovascular Surgery [1].

Prior to that groundbreaking publication, it was already recognised that a group of malformations had comparable morphology comprising abnormal atrioventricular valves. Blieden and colleagues [2] had previously described disproportion between the inlet and outlet dimensions of the ventricles, readily demonstrated by the left ventricular ‘goose-neck’ on angiography, as the key to diagnosis. At that stage most described the group of lesions as ‘atrioventricular canal malformation’ or ‘endocardial cushion defect’ [3,4]. Perhaps more significantly, the so-called ostium primum defect was thought to represent a partial variant. It was also universally thought, at that time, that the left atrioventricular valve was no more than a mitral valve with a cleft in the aortic leaflet. In addition to this, lesions such as isolated cleft of the mitral valve, large ventricular septal defects opening to the inlet of the right and hearts with straddling or overriding tricuspid valve were considered variations of the atrioventricular canal malformation.

There were two extremely important events contributing to the breakthrough in understanding. The first was when Anderson and Becker, working with heart specimens in Amsterdam, stripped away completely from the atrioventricular junction, the leaflets of the atrioventricular valves. Having removed the valve leaflets, it was realised that in any individual heart with this group of anomalies, it was impossible to know whether, initially, the specimen had represented a so-called ‘partial’, ‘intermediate’ or ‘complete’ example of the anatomy, because each of the hearts had a common atrioventricular junction completely different from the normal heart. The second important event was when cardiologists, using their newly acquired skills in cross-sectional echocardiography, were able to produce exquisite moving images of these hearts, representing cross-sectional anatomic sections. I recall Bob Anderson being ‘blown away’ by what he was seeing at our regular weekly meetings and immediately our understanding began to reach completion. With morphological sections of the heart [5], he simulated echocardiographic sections in four chamber and short axis of the atrioventricular junction, confirming, at a stroke, these provided almost all of the information for accurate diagnosis apart from demonstrating the ventricular outflows.

**Figure 1:**
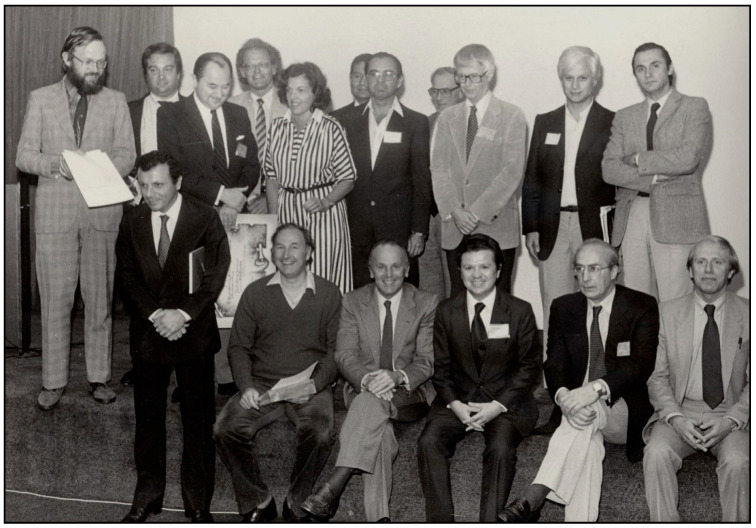
Invited faculty, Brazilian Congress of Pediatric Cardiology, Porto Alegre, 1982.

By coincidence, in 1982 we were both guests of the Brazilian Society of Pediatric Cardiology in Porto Alegre around the time of publication. During this, my first visit to South America (Figure 1) it was no coincidence that some of the academic sessions revolved around the potentially controversial topic of AVSD, the subject of the ‘What’s in a name’ publication. Anderson and Becker had advocated dismissing the terms ‘endocardial cushion defect’ and ‘atrioventricular canal defect’ on the basis they were morphologically unsound and inaccurate despite them being in common usage. By that time, I had found their arguments persuasive and, using the ‘new’ nomenclature, together we demonstrated heart specimens and moving echocardiographic images of the heart painstakingly recorded and edited onto high quality video tape. The presentations and recommendations were received enthusiastically by the large international faculty and many delegates. So began a new era of understanding, crucial to improvements in surgical outcome. However, although it was the intention of Becker and Anderson to provide a precise and accurate way of describing hearts with AVSD, the term has continued to be used imprecisely by many cardiologists and surgeons, as I will outline later.

## 2. Consideration of the Atrioventricular Junction and Valve Leaflets

**Figure 2:**
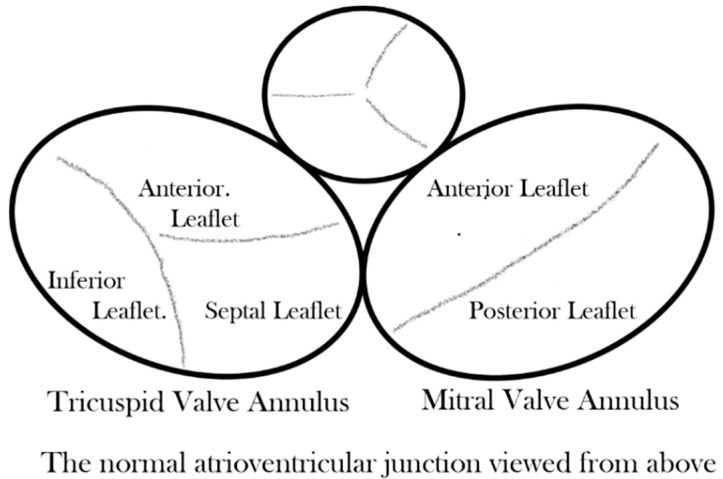
Drawing of the normal atrioventricular junction.

Considering first the normal atrioventricular junction [6], it is divided into discrete right and left components that surround the orifices of the mitral and tricuspid valves, respectively (Figure 2). The two junctions are themselves contiguous only over a very short area, which is that of the muscular atrioventricular septum anterior, and superior to this short area, the subaortic outflow tract of the left ventricle interposes between the mitral valve and the muscular ventricular septum and the fibrous membranous septum. At all other points around the atrioventricular injunctions, the fibrofatty tissue of the atrioventricular grooves interposes between atrial and ventricular myocardium and becomes contiguous with the fibrous leaflets of mitral and tricuspid valves while the aortic valve is wedged anteriorly between the mitral and tricuspid valves.

In essence, Anderson and Becker had proposed the term ‘atrioventricular septal defect’ as a generic name for a group of anomalies characterised by absence of atrioventricular septal structures and consequently possessing a common atrioventricular junction guarded by a common atrioventricular valve and therefore completely different from the atrioventricular junction of the normal heart [7]. There is not only a grossly abnormal atrioventricular junction morphology but also of necessity an abnormally positioned conduction axis as well as the subaortic outflow no longer being able to interpose between the left and right sides of the junction.

**Figure 3:**
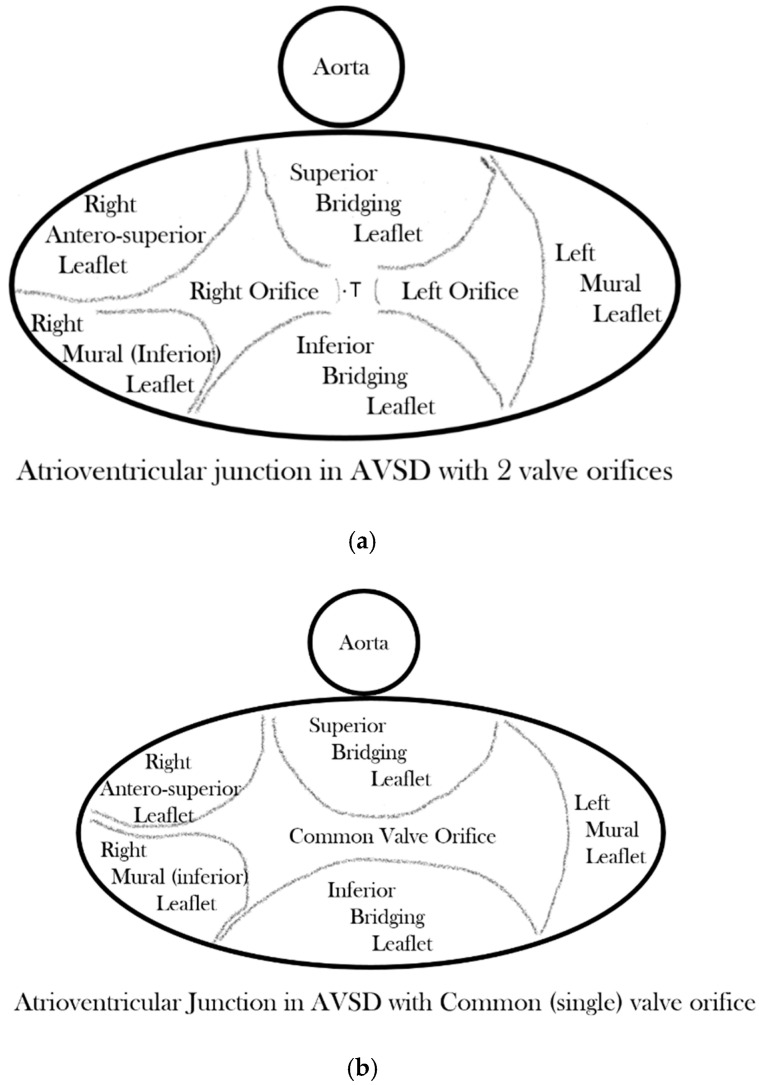
(**a**) Drawing of the atrioventricular junction in Atrioventricular Septal Defect (AVSD) with two valve orifices. (**b**) Drawing of the atrioventricular junction in AVSD with common valve orifice.

In almost every form, the common atrioventricular valve is composed of five leaflets of which only the superior and inferior bridging leaflets are found within both the left and right ventricles, whereas the left mural leaflet is confined to the left ventricle and the right (inferior) mural and right anterosuperior leaflets are located inferiorly and antero-superiorly, respectively, in the right ventricle. The zone of apposition of the inferior and superior bridging leaflets is the most frequent site of valve insufficiency. This common valve will have a single (‘common’) orifice or will be divided into two orifices by a tongue of valve tissue joining the bridging leaflets; this provides the basis for a broad classification of all cases (Figure 3a,b). Irrespective of common or separate orifices the left ventricular component of the common valve has a trileaflet arrangement that cannot be compared with the normal mitral valve, an appellation that should be avoided. The right ventricular component of the common valve similarly should not be called ‘tricuspid’ [6]. It is also important to be aware that ‘AVSD’ on its own is not a diagnosis and should not be used alone to describe any of the various forms of this group of anomalies because it lacks any specificity whatsoever.

With two valve orifices the most frequent form is the isolated primum defect, which is also commonly described as ‘partial AVSD’, although there is nothing partial about the common atrioventricular junction, which is just as complete as that seen in patients with common valve orifice. Other variations include an isolated interventricular defect or combined primum interatrial defect and interventricular component; both forms can also be described as ‘intermediate’, but qualifying additional description is required in diagnosis to distinguish the two forms. Rarely are completely intact septal structures found, an AVSD without interatrial or interventricular communication. With common orifice the main variation is found in the degree of bridging of the superior bridging leaflet, but there is almost always an interatrial and interventricular communication. Whatever the type of AVSD, the left ventricular outflow is sometimes longer than that of the normal heart [8], and the value of the inlet/outlet dimensions ratio was significantly less in hearts with atrioventricular septal defects, with *p* < 0.001 in Table III of that publication. So, it seems to me that it could indeed be a diagnostic criterion on its own.

## 3. Variations from Usual Forms of AVSD

Quite independent of these various types, associated anomalies can include left or right ventricular inflow obstruction, dual orifice left ventricular atrioventricular valve leaflets, atrioventricular valve regurgitation, left or right ventricular outflow tract obstruction in various forms, and right or left ventricular hypoplasia (‘ventricular imbalance’). Left ventricular outflow obstruction can be particularly complex. The subaortic outflow is frequently elongated and relatively narrow in all forms of AVSD. The causes of obstruction include anomalous tissue tags, anomalous chords to the ventricular septum, discrete fibromuscular stenosis and tunnel-like narrowing. Another variation to be aware of is double outlet right atrium, in which inevitably blood flows directly from right atrium to left ventricle, causing unexplained systemic arterial desaturation. There is also a well-recognised association of AVSD with Trisomy 21, atrial isomerism, total anomalous pulmonary venous connection and Tetralogy of Fallot and, rarely, even with common arterial trunk.

The key to the diagnosis of AVSD and, to a major extent, appropriate and effective surgical management lies in the structure of the atrioventricular valve leaflets found in the left ventricle [9,10]. When the left atrioventricular component of the common junction is considered in isolation, the mural leaflet forms less than one-third of the circumference of the orifice, the remainder being formed of components of the superior and inferior bridging leaflets, which together form a zone of apposition that some still and incorrectly describe as ‘a cleft’. Even if a surgeon were to suture together completely this zone of apposition, such a manoeuvre would not restore the morphology to that of a normal mitral valve. There is not complete agreement as to whether or not at the time of operation the surgeon should suture together, partially or completely, the zone of apposition, particularly in the absence of preoperative valve insufficiency.

There is a potentially complicating aspect when the left ventricular valve leaflets lack the usual morphology found in an AVSD, and this also has major surgical significance [11,12]. The classical distortion is found in parachute malformations. In such cases all the valve chords attach to a single papillary muscle, usually because the mural leaflet is extremely small or absent. The valve orifice is then represented only by the zone of apposition between the bridging leaflets, and the key point of surgical significance is it cannot be sutured, even in the presence of significant valve insufficiency, because stenosis will almost inevitably be the outcome. However, the parachute malformation of the left AV valve in AVSD may not be caused by an absent mural leaflet. It could be the reverse in that, if there is a single papillary muscle, then the mural leaflet is by necessity absent. In the study by Oosthoek PW et al. [13], the anomaly of the papillary muscles was considered the primary event leading to parachute valve. Other deviations from the usual valve morphology include leaflet dysplasia, a small or miniaturized orifice, short superior bridging leaflet, extremely short valve chords or dual orifice produced by an anomalous bridge of valve tissue between any two of the leaflets.

It is important briefly to discuss a rare form of deficient atrioventricular septation with separate left and right atrioventricular junctions. The patients with deficiency of the fibrous atrioventricular septum, although properly described as atrioventricular septal defects, do not have a common atrioventricular junction. It was the view of Anderson that it was more convenient to describe them as ‘Gerbode’ defects while recognising their affinity with hearts with perimembranous VSD combined with the potential for ventriculo-atrial shunting because of a deficiency in the commissure between the septal and anterior leaflets of the tricuspid valve.

## 4. Diagnostic Considerations

The diagnosis of any form of AVSD is almost always made by cross-sectional echocardiography, which provides exquisite imaging and a precise diagnosis in most cases, particularly in early life [5,7]; magnetic resonance imaging, rarely required in primary diagnosis, can also be helpful and carries the advantage of being able to demonstrate the atrial and ventricular components en face more reliably than transthoracic three-dimensional echocardiography in older patients. In practice, because primary diagnosis is made in infancy, the resolution of 3D echocardiography makes it of limited value in presurgical work up. But cardiac ultrasound does allow a complete morphological diagnosis in most cases, and colour flow Doppler is complementary because it shows the sites of intracardiac shunting and the location and direction of any atrioventricular valve regurgitation. There is frequently a curtain of regurgitant blood flow between the bridging leaflets, and although exact quantification of valve insufficiency is often difficult, it can broadly be classified into trivial, mild, moderate or severe. Atrioventricular septal defects may be encountered when there is atrial isomerism, and for this reason the accurate determination of atrial arrangement, atrioventricular and ventricular relationships, ventricular topology, great artery relationships and systemic and pulmonary venous drainage is also important. The various forms of AVSD are now readily diagnosed antenatally by the fetal cardiologist; an accurate description and complete diagnosis also provides important information regarding postnatal prognosis.

**Figure 4:**
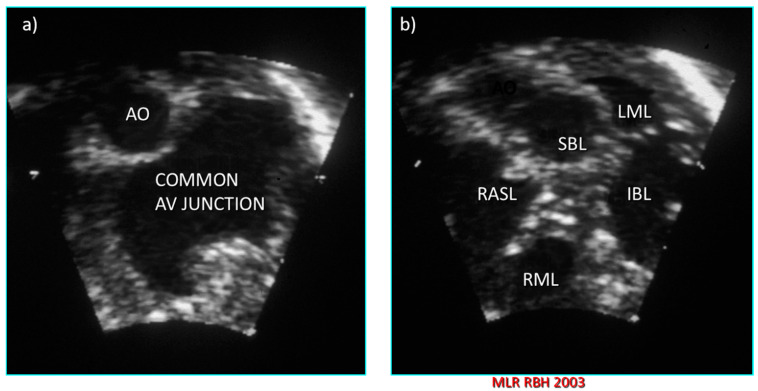
Subcostal left oblique short axis echocardiographic sections of the AV junction showing (**a**) the common atrioventricular junction and (**b**) the five leaflets of the common atrioventricular valve.

**Figure 5:**
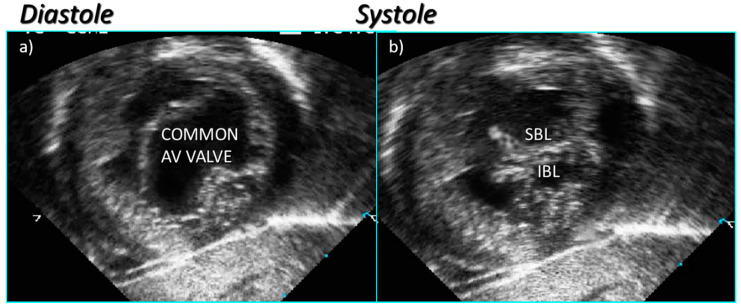
Subcostal left oblique short axis echocardiographic section of AV junction at level of AV valve leaflets showing (**a**) common atrioventricular valve during atrial systole and (**b**) the zone of apposition of the superior and inferior bridging leaflets.

**Figure 6:**
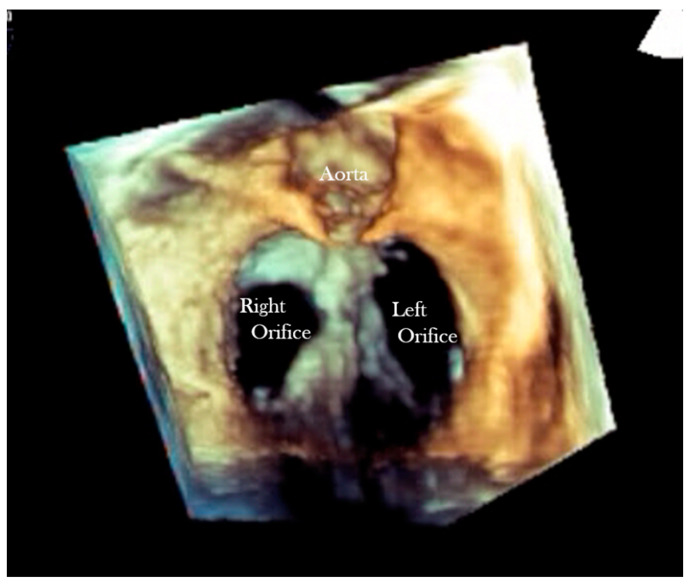
Short axis 3D echocardiographic section in a heart with AVSD and two valve orifices.

**Figure 7:**
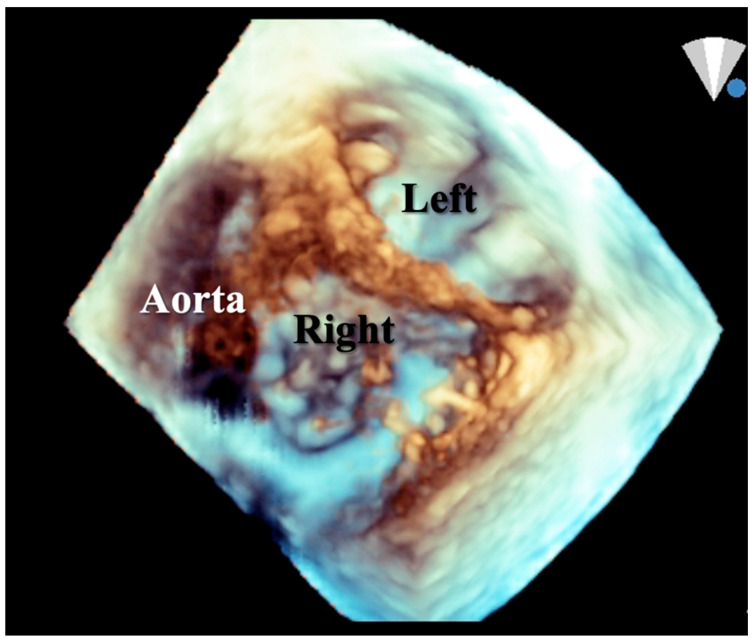
Short axis 3-D echocardiographic section in a heart with partial AVSD with left and right valve orifices. Part of the ventricular septum can be seen between these orifices.

**Figure 8:**
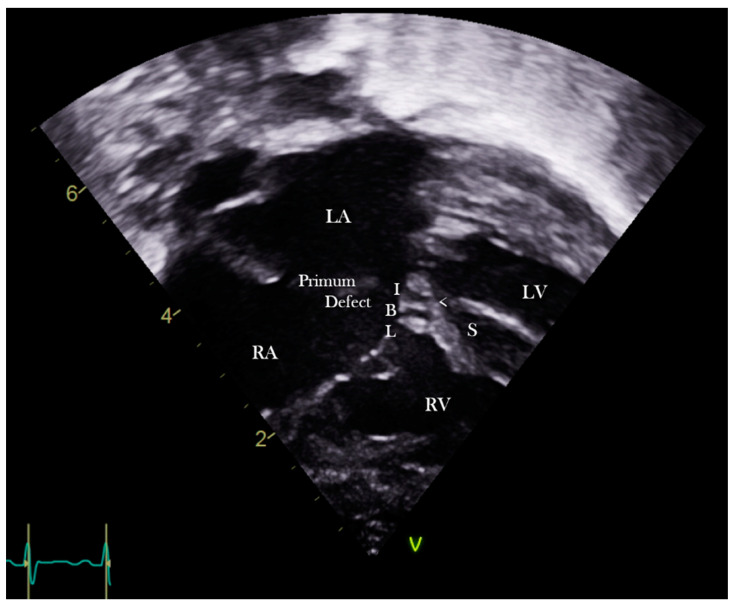
Subcostal echocardiographic four chamber section showing the inferior bridging leaflet.

**Figure 9:**
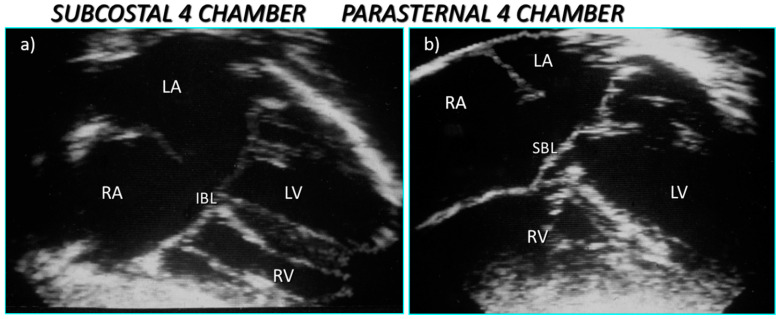
Echocardiography sections from a partial atrioventricular septal defect showing (**a**) the primum defect and inferior bridging leaflet in the subcostal section and (**b**) the anterior and bridging leaflet in the apical section with an obvious recess beneath the superior bridging leaflet.

There are some basic rules of cross-sectional echocardiography that are fundamental to the diagnosis [5,7]. The subcostal short axis section can always demonstrate a common atrioventricular junction guarded by a common atrioventricular valve with the aortic valve in an anterior and ‘unwedged’ position (Figure 4 and Figure 5). These features can also be demonstrated by 3D echocardiography (Figure 6 and Figure 7). This seems to be a consistent and diagnostic echocardiographic feature of all atrioventricular septal defects that can be equally well seen with magnetic resonance imaging. In many instances both imaging techniques potentially allow the differentiation of each of the five leaflets of the common atrioventricular valve. In general terms echocardiographic four chamber sections allow the immediate recognition of an atrioventricular septal defect. It is important to be aware, however, that it is the subcostal four chamber section used expertly that best defines the inferior bridging leaflet (Figure 8), clearly inferior to the more anterior, superior bridging leaflet (7). When scanning from the horizontal subdiaphragmatic sections to the paracaronal section of the atrioventricular junction, it is the inferior leaflet that is imaged first. The apical and parasternal four chamber sections allow visualisation of the more anterior, superior bridging leaflet (Figure 9). The subcostal long axis section will show the left ventricular outflow tract formed medially by the superior bridging leaflet and laterally by the wall of the left ventricle. The trileaflet left atrioventricular valve will be evident on subcostal and parasternal short axis sections of the left ventricle. This is again a very consistent finding in atrioventricular septal defects and is particularly useful in unusual examples when standard four chamber sections are not diagnostic. The trileaflet valve is composed of the left mural leaflet and the left ventricular components of the superior and inferior bridging leaflets. The commissure between the bridging leaflets that points towards the ventricular septum is still often described incorrectly as a ‘cleft’.

It is important to be aware that the typical trileaflet appearance of the left ventricular valve is distorted when there is a dual orifice or severe leaflet dysplasia [10]. A two-leaflet valve will be found when the left mural leaflet is absent, and this can result in problems in diagnosis of an AVSD, particularly in cases with an isolated interventricular defect or intact septal structures. However, the bileaflet valve is still quite distinct from the normal mitral valve. Absence of the left mural leaflet frequently causes the left-sided AV valve to be small and or potentially stenotic. As I described earlier, a parachute abnormality is also associated with a bileaflet valve, and short axis echocardiographic sections of the left ventricle will usually reveal a single papillary muscle.

**Figure 10:**
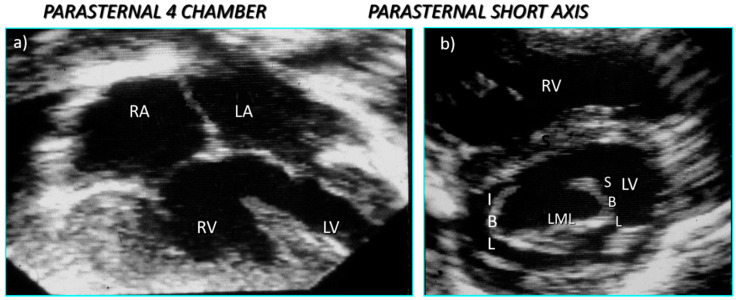
Echocardiographic sections from a heart with the form of AVSD in which (**a**) the parasternal four chamber section reveals the isolated interventricular communication and (**b**) the parasternal short axis section reveals the trileaflet left atrioventricular valve.

The mainstay of diagnosis remains cross-sectional echocardiography [5,7]. The most frequently observed form of an atrioventricular septal defect with two valve orifices is an isolated primum defect (‘partial AVSD’) between the lower edge of the atrial septum and the atrioventricular valve leaflets (Figure 9). In four chamber sections there appear to be two atrioventricular valves, each attached to the crest of the ventricular septum and in continuity through the bridging leaflets, so that there is absence of the normal offsetting. The commonly observed recess, directly below the superior bridging leaflet, attached to the ventricular septum should not be described as an interventricular communication or VSD. Less common variants with two valve orifices include complete absence of a primum defect (‘intermediate AVSD’), with an interventricular communication masquerading as a perimembranous inlet ventricular septal defect (Figure 10a,b); the diagnosis relies on the finding of a trileaflet left atrioventricular valve (Figure 10b) and a common atrioventricular junction. Alternatively, both a primum defect and an interventricular component can be seen in parasternal four chamber sections. The ventricular component is then usually relatively small, and this variation has also been called an ‘intermediate’ form. Rarely, an isolated trileaflet left atrioventricular valve may be associated with intact septal structures, although in a number of cases spontaneous closure of a small interventricular component will have occurred postnatally. Nevertheless, it is important to be aware that an atrioventricular septal defect can occasionally exist in the absence of any atrial septal defect or interventricular communication. We have encountered such cases presenting with severe left atrioventricular valve regurgitation, but it was usually possible for the valve to be repaired at surgery. These cases also need to be distinguished from the isolated cleft of the anterior leaflet of the mitral valve, which is directed towards the outlet of the left ventricle rather than towards the septum.

**Figure 11:**
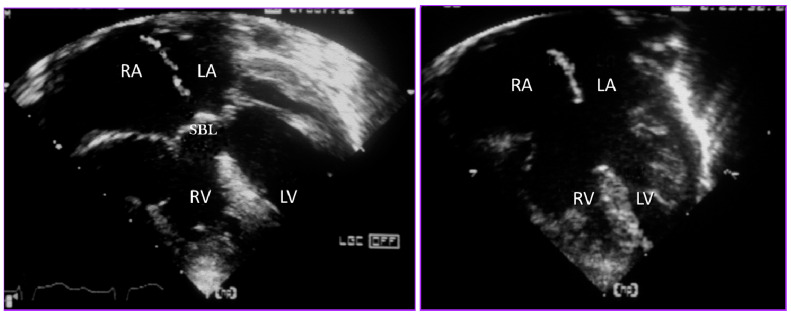
Echocardiographic sections from a heart with complete AVSD (**a**) during ventricular systole with the valve closed and (**b**) during diastole with the valve open.

The typical features of the so-called ‘complete’ AVSD are also best identified in four chamber sections and include a primum interatrial defect, common atrioventricular valve orifice and an interventricular communication that can be more or less extensive, depending on the attachment of bridging leaflets (Figure 11). The inferior bridging leaflet is frequently attached by a mid-line raphe to the septal crest so that there is no additional defect posteriorly, but it may be free-floating so that the defect extends beneath this leaflet. Most variation in the size of the ventricular septal component, however, is found anteriorly beneath the superior bridging leaflet and is evident on parasternal four chamber sections. There is considerable variation in the amount of bridging of the superior leaflet, which may be more or less extensive. Extreme bridging is more likely to be encountered in those cases associated with Tetralogy of Fallot or double outlet right ventricle.

## 5. What Is in a Name?

So, what is in a name when today’s cardiovascular imaging is so good? In many situations, the exquisite detail provided by echocardiography cannot be described in a single word or phrase, although in most cases there is a primary diagnosis that alerts physicians and surgeons to the basic abnormality [14]. Considering first the simplest form of atrioventricular septal defect, the commonly used term ‘primum atrial septal defect’ or ‘partial AVSD’ is clearly preferable to ‘atrioventricular septal defect with two valve orifices and an isolated interatrial defect’, if only because the former is shorter and more precise and everyone knows what it means. Anderson and Becker understandably disliked the term ‘partial’ because there is a complete common atrioventricular junction. Of course, there may be the additional abnormalities I referred to earlier as well as atrioventricular valve regurgitation or left ventricular outflow tract obstruction, but these can be included as supplementary lines in the diagnosis, enabling a full description of the heart to emerge.

But what of hearts with a common atrioventricular junction and common orifice (‘complete AVSD’)? Such examples are often called ‘atrioventricular septal defect’ as the primary diagnosis. This is clearly an unsatisfactory state of affairs taking into account that this is the selected generic name for all types, but the terms ‘complete atrioventricular septal defect’ or even ‘complete atrioventricular canal defect’, although imperfect, are still better able to convey a diagnosis that everyone understands. To my mind ‘atrioventricular septal defect with common valve orifice’ is too much of a mouthful. Any additional abnormalities such as left or right ventricular hypoplasia (‘ventricular imbalance’) can be recorded as additional secondary diagnoses.

So, what should we call the various types of AVSD? Always, the underlying principle is precise and accurate communication. I would suggest ‘complete AVSD’ is a term understood and accepted as referring to hearts with a common valve orifice and primum and interventricular defects. I would also advocate that the terms ‘partial’ or ‘incomplete’ refer to all types of AVSD with two valve orifices and isolated primum defect while ‘intermediate’ refers to any AVSD with two valve orifices and an interventricular communication. However, in the nomenclature of CHD (congenital heart disease) recently designed by the ISNPCHD for the ICD-11 [15], the term ‘intermediate AVSD’ (and its synonym ‘transitional AVSD’) has a very different meaning. It refers to an “Atrioventricular septal defect with communication at atrial level and restrictive communication at ventricular level”, when there is complete absence of a primum defect (‘intermediate AVSD’) corresponding in ICD-11 to an “Atrioventricular septal defect with communication at the ventricular level only”, with the synonym “atrioventricular septal defect with isolated ventricular component”. It is therefore better to be more specific and use the following terms:

- ‘Isolated primum ASD’ or ‘partial AVSD’,
- ‘AVSD with two valve orifices and isolated ventricular component’ or ‘AVSD with isolated VSD’,
- ‘AVSD with two valve orifices, primum ASD and small ventricular component’ or ‘intermediate AVSD with primum ASD and VSD’,
- ‘Complete AVSD’,
- ‘AVSD with intact septal structures’.

This basic classification can be used when there are deviations from the usual forms, such as ventricular hypoplasia [12,14,15,16].

**Figure 12:**
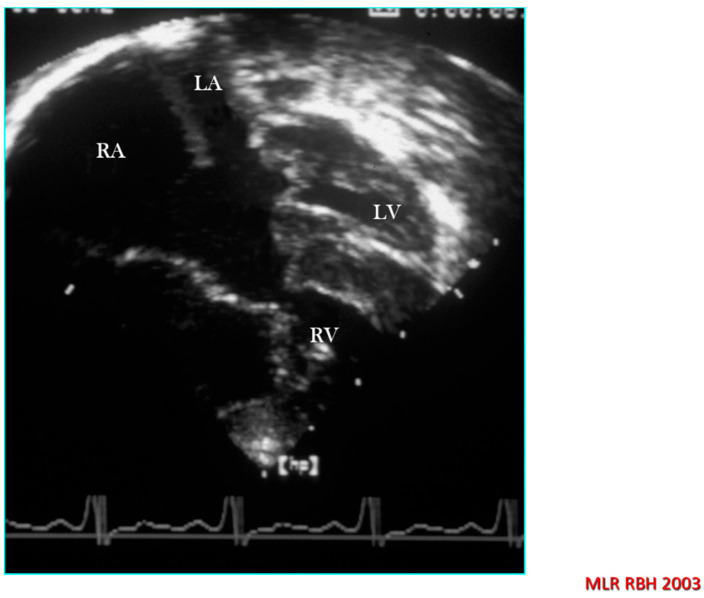
Echocardiographic four chamber section from a heart with partial AVSD, hypoplastic left ventricle and miniaturised left atrioventricular valve orifice.

Problems in nomenclature can also arise in some hearts with AVSD because there is a profound departure from the usual morphology. For example, with usual atrial arrangement, if the atrioventricular connection is discordant, then the ventriculo-arterial connection is almost always discordant or double outlet right ventricle. The diagnosis requires the accurate recognition and description of ventricular morphology and ventricular topology, but the unifying feature will remain a common atrioventricular junction albeit with the right side of the common valve having a trileaflet structure instead of the left. Similar ventricular relationships may be encountered with atrial isomerism and atrioventricular septal defect in Figure 12 [16]. When there are abnormalities of atrioventricular connection, the essential role of imaging is a sequential segmental approach to diagnosis in order to fully describe such hearts. In such a situation a name is unimportant, but describing the way the atria connect to the ventricles and the morphology of the atrioventricular valve together with details of the type and mode of ventriculo-arterial connection is crucial to accurate diagnosis and surgical planning.

## 6. Other Examples of Absence of the Atrioventricular Septum

**Figure 13:**
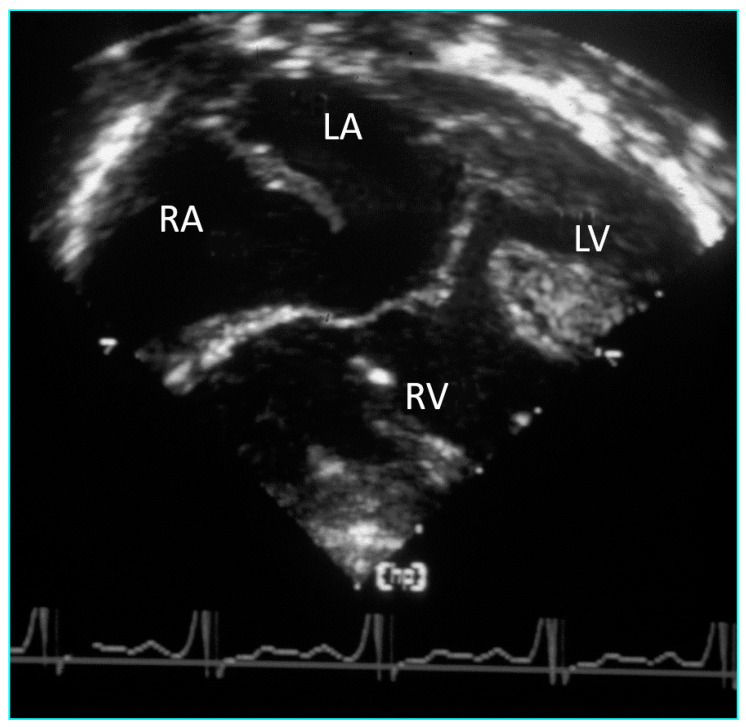
Echocardiographic four chamber section from a heart with complete AVSD, hypoplastic left ventricle and the morphological criteria for the double inlet right ventricle.

**Figure 14:**
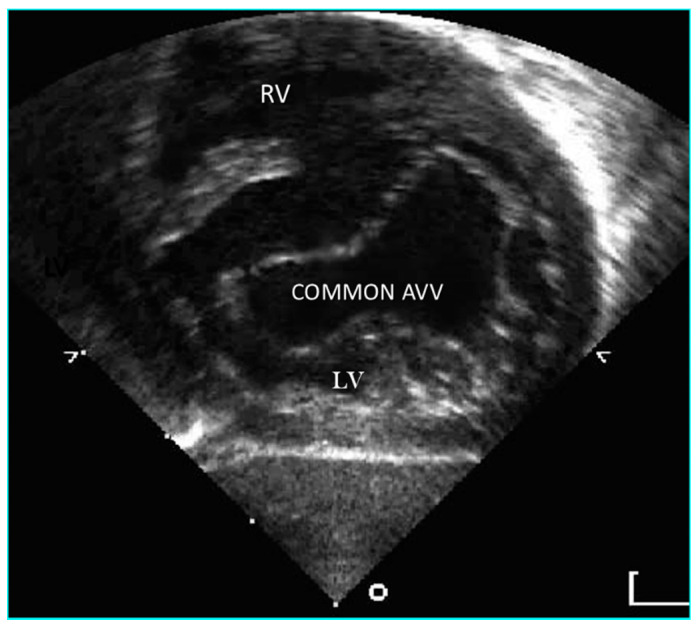
Subcostal left oblique echocardiographic section at the level of the atrioventricular junction showing the common atrioventricular valve committed entirely to the left ventricle.

At the outset I explained that according to Anderson and Becker one of the hallmarks of an AVSD was absence of the atrioventricular septum. Yet they would accept that this will be absent in other situations such as tricuspid atresia, double inlet left ventricle with two atrioventricular valves and even some posterior ‘inlet’ perimembranous ventricular septal defects. These malformations, however, fail to satisfy a second criterion for inclusion, a common atrioventricular junction. But there is a close cousin, which, although characterised by absence of the atrioventricular septum and a common atrioventricular junction, is not necessarily an atrioventricular septal defect. In some cases of double inlet ventricle, a common atrioventricular valve neither straddles nor overrides the ventricular septum and is committed entirely to one ventricle. Such hearts are not examples, therefore, of an atrioventricular septal defect with ventricular imbalance but clear examples of a univentricular atrioventricular connection with double inlet. Even those who might argue the case for using the term unbalanced AVSD when there is a dominant left or right ventricle could not continue the argument in the presence of double inlet connection to a solitary indeterminate ventricle. It is, however, morphologically correct to describe the connection as double inlet when 75% or more of the common atrioventricular valve is committed to one ventricle in the presence of a second hypoplastic and rudimentary contralateral ventricular chamber (Figure 13 and Figure 14). I recall Anderson informing me “we have thus far been unable to discern any consistent difference in the characteristics of the common valve in double inlet and atrioventricular septal defect although in the former the common valve sometimes consists of four rather than five leaflets”.

## 7. Some Implications of Names and Classification

Finally, I will put forth the question, ‘What is in a name, particularly when today’s imaging techniques show everything’? The fundamental reason for allocating names to congenital heart malformations is to allow communication between those working in the field. Names are also essential to research and audit. Surgeons must be able to investigate the results of radical repair of hearts with the various morphological features of an atrioventricular septal defect and identify any incremental risk factors for poor outcome. Retrospective review can only be made possible by an adequate system of nomenclature and classification.

Learning to be a paediatric cardiologist or cardiac surgeon is extremely complex. For example, to the less experienced, echocardiographic, angiographic, CT or resonance images of the heart appear at first as a meaningless blur. Gradually, however, our brains are able to connect together the findings on clinical examination of the patient with the images that we see. For example, even without allocating a name, an echocardiographic four chamber section from a patient with a primum atrial septal defect will immediately trigger a variety of complex thoughts ranging from expectations of clinical findings, symptoms, natural history and likely outcome of repair. The surgeon will imagine the appearance of the heart in the operating room while the morphologist will be aware of what the heart would be like held in the hand and examined in the laboratory. For the individual expert, names have potentially become irrelevant. For those of us involved in managing patients with congenital heart disease it is often unwise to rely entirely on individual names for particular conditions because of the presence of unusual features or associated anomalies.

**Figure 15:**
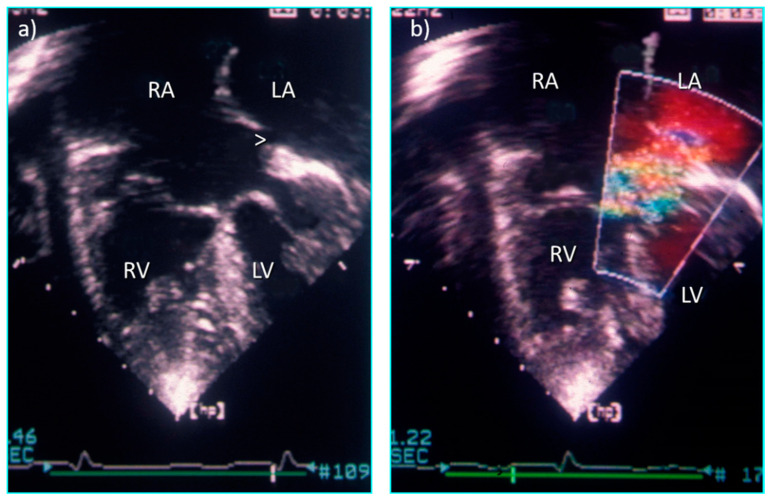
Four chamber echocardiographic sections from a heart with double outlet right atrium and restrictive primum defect (ASD) (**a**) without colour flow Doppler and (**b**) with turbulence across the restrictive ASD.

Consider the hypothetical situation of a paediatric cardiologist who, on the basis of a cross-sectional echocardiogram, informs a cardiac surgeon about a patient with a primum atrial septal defect that requires routine repair, and the surgeon takes the patient to the operating room without carefully reviewing and discussing any investigations. For a simple primum defect, no doubt the outcome would be very good because, simply on the basis of the name, the surgeon would anticipate correctly the operative findings. But just imagine the likely consequence if in addition to the primum defect there had been double outlet right atrium, a small and restrictive primum defect with left atrial hypertension, double orifice left atrioventricular valve and left ventricular outflow tract obstruction (Figure 15) caused by anomalous chords from the superior bridging leaflet to the ventricular septum. The patient would have been much more likely to survive the operation or have a perfect outcome of repair had the surgeon undertaken a complete review of all the investigations and completely understood the data. But giving the cardiac malformation a ‘label’ is unlikely materially to improve outcome further if the cardiac surgeon was unable to interpret echocardiographic or magnetic resonance images and relied entirely on verbal or written descriptions of any anomalies and observations at the time of surgery.

Consider also another hypothetical situation of a four-month-old infant with isolated primum defect, mild to moderate left atrioventricular valve regurgitation with unexpected congestive heart failure and failure to thrive. The cardiologist was unable to be present when the case was shown to colleagues and surgeons, and an echocardiogram was interpreted as a straightforward ‘partial AVSD’ requiring early surgical repair and the management was agreed upon quickly. At operation the surgeons found an atypical left atrioventricular valve and could not achieve a complete operation. In a retrospective review, the outpatient report from the referring cardiologist was discovered.

‘Partial Atrioventricular Septal Defect (Isolated primum ASD)

Bileaflet left atrioventricular—deficient left mural leaflet

Parachute deformity of left AV valve

Small/potentially stenotic left-sided valve orifice

Short chords of left-sided valve orifice

(Repair of left ventricular AV valve may be impossible)’

This is an example of a case where access to the complete diagnosis at the multidisciplinary team meeting and a more careful review of the clinical history and the echocardiogram at the time would have alerted everyone to potential difficulties and possibly changed the clinical management and surgical approach and timing.

Given the precision and quality of cardiovascular imaging now available and the ability to transmit digital images with great speed virtually anywhere, it is difficult to avoid the conclusion that names are less important than they were. We must not forget, however, that words, names and language are the essential prerequisites of communication, audit and teaching. Good practice in the management of congenital heart disease cannot be achieved without accurate communication! A complete and accurate description in the diagnosis will also alert the surgeon to potential difficulties that will be encountered during surgery, and, as I have tried to convey, there can be a complex constellation of deviations from the usual morphology. Indeed, I have also attempted to emphasise both the clinical (surgical) and scientific importance of preciseness. Preciseness, no matter how impractical it may be in contemporary ‘fast-track’ life and medicine, implies a deep understanding, which is a hallmark of the Anderson and Becker school of morphology. The marvellous contribution of Bob Anderson and his many collaborators to the understanding of congenital heart malformations has in part been made possible by the emphasis on nomenclature and precise communication. However, it is regrettable that his message seems not yet to have reached everyone.

## Data Availability

Not available.

## Abbreviations

RA	right atrium
LA	left atrium
RV	right ventricle
LV	left ventricle
S	ventricular septum
LML	left mural leaflet
SBL	superior bridging leaflet
RASL	right antero-superior leaflet
RML	right mural leaflet
IBL	inferior bridging leaflet
T	Connecting Tongue of valve tissue
AVV	atrioventricular valve
Ao	Aorta
